# Efficacy and safety of soluble guanylate cyclase stimulators or activators for chronic kidney disease: a systematic review and meta-analysis

**DOI:** 10.3389/fmed.2026.1778037

**Published:** 2026-03-02

**Authors:** Jiaying Zhang, Xin Li, Xiaofeng Yu

**Affiliations:** 1 Department of Nephrology, The Third Hospital of Mianyang/Sichuan Mental Health Center, Mianyang, Sichuan, China; 2 Department of Neurosurgery, Chengdu Third People’s Hospital, Chengdu, Sichuan, China; 3 Department of Cardiology, The Third Hospital of Mianyang/Sichuan Mental Health Center, Mianyang, Sichuan, China

**Keywords:** chronic kidney disease, meta-analysis, systematic review, soluble guanylate cyclase activators, soluble guanylate cyclase stimulators

## Abstract

**Background:**

Chronic Kidney Disease (CKD) poses a major global health burden, leading to serious complications and death. The nitric oxide (NO)-soluble guanylate cyclase (sGC)-cyclic guanosine monophosphate (cGMP) signaling axis regulates various kidney functions. sGC stimulators (sGCs) and activators (sGCa) are emerging as a potential new approach for the treatment of renal disorders. However, there is still a lack of large-scale research on CKD.

**Methods:**

We systematically searched the PubMed, Embase, Web of Science, and Cochrane Library databases from January 1971 to December 2025 to identify studies examining the effects of sGCs or sGCa on CKD. Pooled standardized mean differences (SMDs) or odds ratios (ORs) with 95% confidence intervals (CIs) were calculated for study outcomes.

**Results:**

Ten studies were included in the final analysis. The administration of sGCs or sGCa was associated with significant reductions in kidney weight (SMD = −1.55, 95%CI: −2.19, −0.90), systolic blood pressure (SMD = −3.52, 95%CI: −6.48, −0.56), and serum uric acid levels (SMD = −3.82, 95%CI: −4.84, −2.80), alongside improved renal function (serum creatinine: SMD = −3.24, 95%CI: −4.94, −1.55; blood urea nitrogen: SMD = −3.53, 95%CI: −5.30, −1.76). However, no significant impact on body weight was observed (SMD = −0.24, 95%CI: −1.17, 0.68). Subgroup analysis indicated that treatment efficacy remained consistent regardless of the specific sGC type but may vary across different forms of chronic kidney disease.

**Conclusion:**

This preclinical meta-analysis indicates that sGC stimulators and activators exert renoprotective effects in CKD, with efficacy potentially influenced by disease etiology. By restoring impaired NO–sGC–cGMP signaling through distinct mechanisms, these agents may offer complementary therapeutic options for different CKD types and inform future clinical trial design.

**Systematic review registration:**

The present study has been registered on PROSPERO (Registration No. CRD420251162902).

## 1 Introduction

Chronic kidney disease (CKD) poses a major threat to global public health and imposes a significant disease burden (1, 2). It is strongly linked to cardiovascular risk, with patient mortality rising as renal function declines (3, 4). Consequently, delaying disease progression and preventing cardiovascular events are central management goals. In terms of pharmacotherapy, the treatment landscape has expanded beyond traditional renin-angiotensin-aldosterone system inhibitors (RASSi) to include newer agents such as sodium-glucose cotransporter-2 inhibitors (SGLT2i) (5), nonsteroidal mineralocorticoid receptor antagonist (nsMRA) (6), and glucagon-like peptide-1 receptor agonists (GLP-1RA) (7), offering more options for CKD management.

Soluble guanylate cyclase (sGC) functions as the pivotal enzyme in the NO-sGC-cGMP pathway (8). Once activated *in vivo*, it produces the second messenger cGMP from GTP. cGMP, in turn, activates multiple downstream effectors, mediating crucial functions including vascular smooth muscle relaxation, suppression of platelet aggregation, and protection against apoptosis and inflammation (9, 10). Based on their mechanism of action relative to the sGC heme group, non-NO-dependent sGC-targeted drugs comprise two classes. sGC stimulators (sGCs) function by binding to and enhancing sGC that contains an active, reduced heme cofactor. In distinction, sGC activators (sGCa) operate independent of the heme moiety and can directly activate sGC in its heme-deficient or oxidized forms (11).

Despite the proven efficacy and established use of sGC-targeted drugs (sGCs/sGCa) in cardiovascular conditions such as heart failure and pulmonary arterial hypertension, their role in CKD remains limited and investigational (12–14). To date, support for their use in CKD comes mainly from preclinical data and small clinical studies. There is still a lack of large-scale research on CKD. This systematic review therefore aims to synthesize and critically appraise the available *in vivo* preclinical evidence to clarify the renoprotective potential of sGCs/sGCa in CKD. Given that current clinical evidence remains limited and primarily exploratory, a focused evaluation of animal studies was undertaken to provide a systematic preclinical foundation for future translational research and the rational design of clinical trials.

## 2 Methods

### 2.1 Search strategy

This systematic review and meta-analysis was conducted and reported in accordance with the PRISMA guidelines, and the study selection process is illustrated using a PRISMA flow diagram.

The search databases for this study were PubMed, Embase, Web of Science, and Cochrane Library from January 1971 to December 2025. The following keywords were used: (Chronic Kidney Disease OR Chronic Kidney Diseases OR Chronic Kidney Insufficiency OR Chronic Renal Disease OR Chronic Renal Diseases OR Chronic Renal Insufficiencies OR Chronic Renal Insufficiency OR disease chronic kidney OR disease chronic renal OR diseases chronic kidney OR diseases chronic renal OR kidney disease chronic OR kidney diseases chronic OR kidney insufficiency chronic OR renal disease chronic OR renal diseases chronic OR renal insufficiencies chronic) AND (soluble guanylate stimulators OR soluble guanylate activators OR riociguat OR vericiguat OR praliciguat OR cinaciguat). To specifically identify preclinical evidence, studies were restricted to *in vivo* animal experiments during the title and abstract screening process, and clinical as well as *in vitro* studies were excluded based on the eligibility criteria. The detailed search strategy is shown in Supplementary Table 1. The present study has been registered on PROSPERO (Registration No. CRD420251162902).

### 2.2 Study selection

The inclusion criteria were as follows: 1. Animal models of chronic kidney disease (all relevant species, i.e., mice and rats) were included, irrespective of the underlying etiology; 2. Treatments included any dose of sGCs/sGCa; controls received placebo or no treatment; 3. The primary outcomes were blood urea nitrogen (BUN) and serum creatinine (Scr); 4. The full text of the study was available in English.

The exclusion criteria were as follows: 1. Non–*in vivo* studies, including *in vitro* experiments, clinical trials, case reports, reviews, editorials, and conference abstracts, were excluded to ensure methodological homogeneity and to focus on preclinical animal evidence; 2. Studies in which the intervention did not involve sGC stimulators or activators were excluded to maintain relevance to the research question; 3. Studies lacking primary outcome measures (BUN and/or serum creatinine) were excluded because these parameters were required for quantitative synthesis and comparability across studies.

Two authors (J. Z and X. L) independently assessed the selected studies for the final analysis, and any discrepancies were resolved through consultation with the third author (X. Y).

### 2.3 Data extraction

Data extraction was performed independently by two authors (J. Z. and X. L.) with predefined forms. The extracted data encompassed study characteristics (e.g., author, publication year, animal model, sample size, CKD induction, intervention, and dose) (Table 1) and primary outcomes [body weight, kidney weight, SBP, SCr, BUN, and serum uric acid (SUA)].

**Table 1:** The basic information table of studies included.

Study	Animal	Number	Induction method	Interventions	Dose	Type of CKD
Chen et al., 2024 (19)	Wistar rats	5/6Nx+HSD+PBO (30) 5/6Nx+HSD+BAY41–8543 (15) 5/6Nx+HSD+BAY60–2770 (15)	5/6 nephrectomized rats on high-salt-diet	sGC stimulators: BAY 41-8543 sGC activators: BAY 60-2770	BAY 41-8543: 2 mg/kg/day BAY 60-2770: 1 mg/kg/day	Hypertensive nephropathy
Abdelrahman et al., 2025 (20)	Wistar rats	Adenine+PBO (6) Adenine+3 mg/kg riociguat (6) Adenine+10 mg/kgriociguat (6)	Adenine-induced chronic kidney disease	sGC stimulators: Riociguat	3 mg/kg/day 10 mg/kg/day	Other types
Wu et al., 2025 (21)	Dahl salt-sensitive rats	Model group (12) Model+HEC-10 (10)	DSS rats on high-salt diet	sGC stimulators: HEC95468	10 mg/kg/day	Hypertensive nephropathy
Al Suleimani et al., 2025 (22)	Wistar rats	CP group (6) CP+3 mg/kg riociguat (6) CP+10 mg/kg riociguat (6)	Cisplatin-caused kidney injury	sGC stimulators: Riociguat	3 mg/kg/day 10 mg/kg/day	Other types
Al-Maskari et al., 2024 (23)	Wistar rats	DX group (6) DX+3 mg/kg riociguat (6) DX+10 mg/kg riociguat (6)	Doxorubicin-induced kidney injury	sGC stimulators: Riociguat	3 mg/kg/day 10 mg/kg/day	Other types
Shea et al., 2020 (24)	Dahl salt-sensitive rats	HS group (8) HS+3 mg/kg praliciguat (8) HS+10 mg/kg praliciguat (8)	DSS rats on high-salt diet	sGC stimulators: Praliciguat	3 mg/kg/day 10 mg/kg/day	Hypertensive nephropathy
Bénardeau et al., 2021 (25)	Sprague–Dawley rats	Ang II+PBO (12) Ang II+2 mg/kg runcaciguat (12) Ang II+6 mg/kg runcaciguat (12)	ANG II induced renal failure	sGC activators: Runcaciguat	2 mg/kg/day 6 mg/kg/day	Hypertensive nephropathy
Atteia et al., 2023 (26)	Wistar rats	Adenine+PBO (10) Adenine+20 mg/kg isoliquiritigenin (10) Adenine+40 mg/kg isoliquiritigenin (10)	Adenine-induced chronic kidney disease	sGC activators: Isoliquiritigenin	20 mg/kg/day 40 mg/kg/day	Other types
Sharma et al., 2025 (27)	db/db mice	db/db+PBO (6) db/db+0.264 mg/kg avenciguat (6) db/db+0.879 mg/kg avenciguat (6)	Diabetic nephropathy	sGC activators: Avenciguat	0.264 mg/kg/day 0.879 mg/kg/day	Diabetic nephropathy
Harloff et al., 2022 (28)	C57BL/6J	dm+PBO (12) dm+cinaciguat (9)	Diabetic nephropathy	sGC activators: Cinaciguat	15 mg/kg/day	Diabetic nephropathy

### 2.4 Quality assessment

We evaluated these studies according to the SYRCLE (Systematic Review Centre for Laboratory Animal Experimentation) standards in ten domains: sequence generation, baseline characteristics, allocation concealment, random housing, blinding of participants and personnel, random outcome assessment, blinding of outcome assessment, incomplete outcome data, selective reporting and other bias (Table 2). Any disagreements were resolved by discussion with a third reviewer (X. Y).

**Table 2:** Quality assessment of studies included by SYRCLE.

Study	Selection bias (sequence generation)	Selection bias (baseline characteristics)	Selection bias (allocation concealment)	Performance bias (random housing)	Performance bias (blinding of participants and personnel)	Detection bias (random outcome assessment)	Detection bias (blinding of outcome assessment)	Attrition bias (incomplete outcome data)	Reporting bias (selective reporting)	Other bias
Chen et al., 2024 (19)	U	L	U	L	L	U	L	L	L	L
Abdelrahman et al., 2025 (20)	U	L	U	L	L	U	L	L	L	L
Wu et al., 2025 (21)	L	L	U	L	L	U	U	L	L	H
Al Suleimani et al., 2025 (22)	L	L	L	L	L	U	L	L	L	L
Al-Maskari et al., 2024 (23)	U	L	U	L	L	U	L	L	L	L
Shea et al., 2020 (24)	U	H	U	L	U	U	L	L	L	L
Bénardeau et al., 2021 (25)	U	L	U	L	L	L	L	U	L	L
Atteia et al., 2023 (26)	U	L	U	L	L	U	L	L	L	L
Sharma et al., 2025 (27)	U	U	U	L	L	L	L	L	L	L
Harloff et al., 2022 (28)	U	L	U	L	L	U	L	L	L	L

### 2.5 Statistical analysis

This meta-analysis was performed using Stata 15.0. All outcomes, treated as continuous variables, are presented as SMDs with 95% CI. Heterogeneity was quantified using the I^2^ statistic, with I^2^ ≤ 50% indicating low heterogeneity and warranting a fixed-effects model, while I^2^ > 50% indicated substantial heterogeneity and justified a random-effects model. Publication bias was assessed using funnel plots and Egger’s test. To explore sources of heterogeneity, subgroup analyses were conducted based on sGC types and CKD induction methods. Sensitivity analysis was performed to evaluate the robustness of the primary outcomes. In this study, *p* < 0.05 indicated that the difference was statistically significant.

## 3 Results

### 3.1 Study inclusion

**Figure 1:**
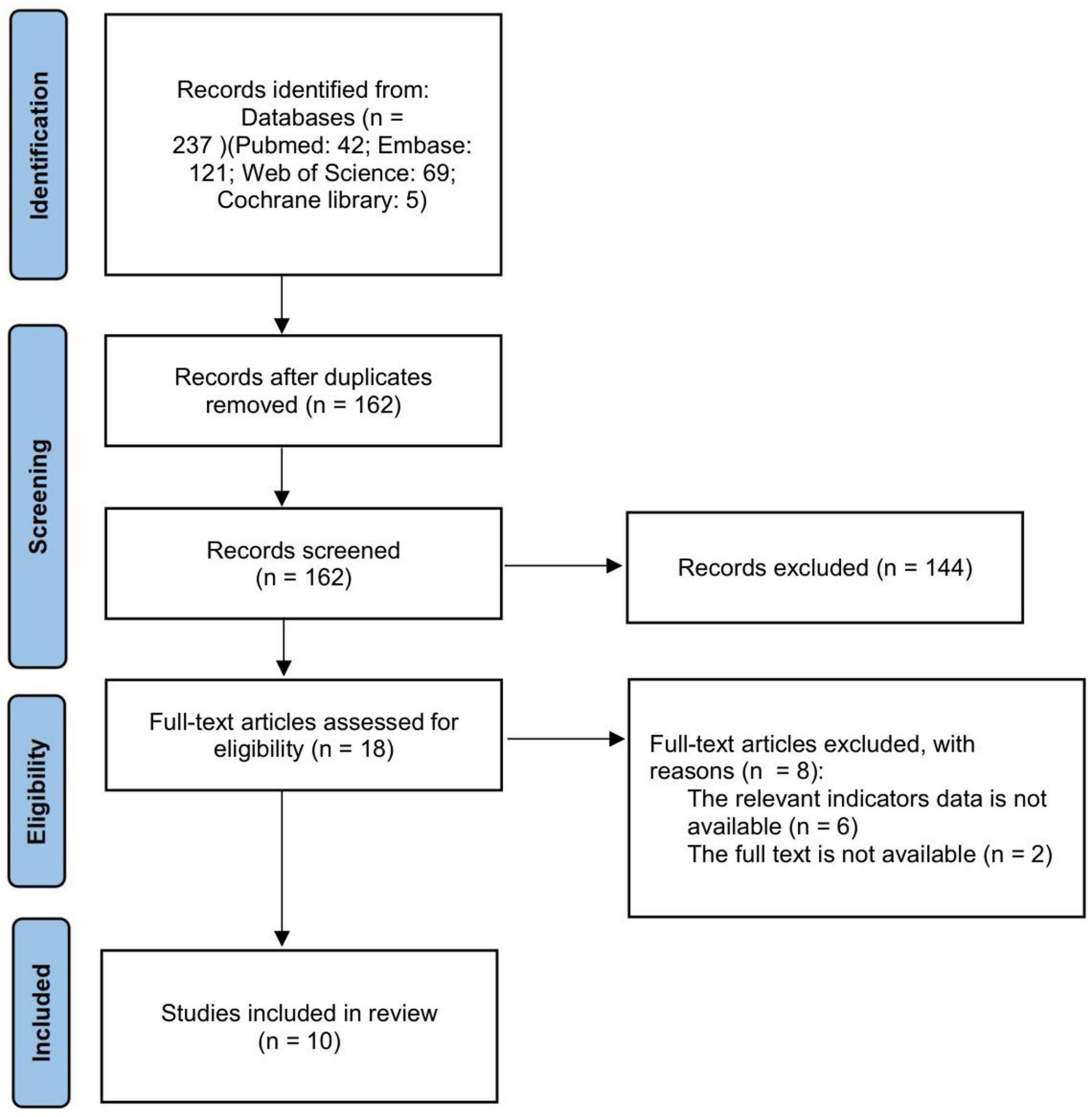
Flowchart of study selection.

Figure 1 depicts the study selection procedure. As a result of the literature search, 237 studies were identified, of which 75 duplicate publications were excluded. After removing duplicates, 162 publications remained, of which 144 were excluded after the title and abstract reading. The remaining 18 articles were further scrutinized by reading the full text. Six studies were excluded because the relevant indicators were not available, and two studies were excluded because the full text was not available.

Ten articles were included (five about sGCs, four about sGCa and one regarding both). Among 10 animal studies, 8 studies used rat, 2 used mice. Two articles focused on diabetic nephropathy, four articles focused on hypertensive nephropathy, and four more articles discussed other types of chronic kidney disease. Basic characteristics of included studies are provided in the Table 1.

### 3.2 Study quality and publication bias

Study quality was evaluated by SYRCLE’s risk of bias tool; a summary of the assessments is provided in Table 2. The primary domains contributing to risk were unclear sequence generation, allocation concealment, and random outcome assessment.

### 3.3 Effects of sGCs/sGCa on CKD

#### 3.3.1 Assessment of body weight and kidney weight

**Figure 2:**
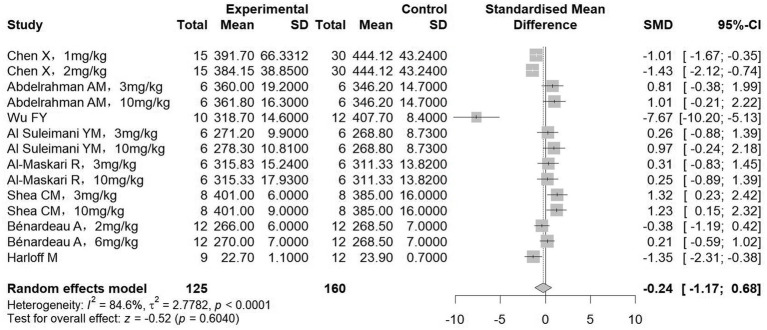
The effect of sGCs/sGCa treatment on body weight.

**Figure 3:**
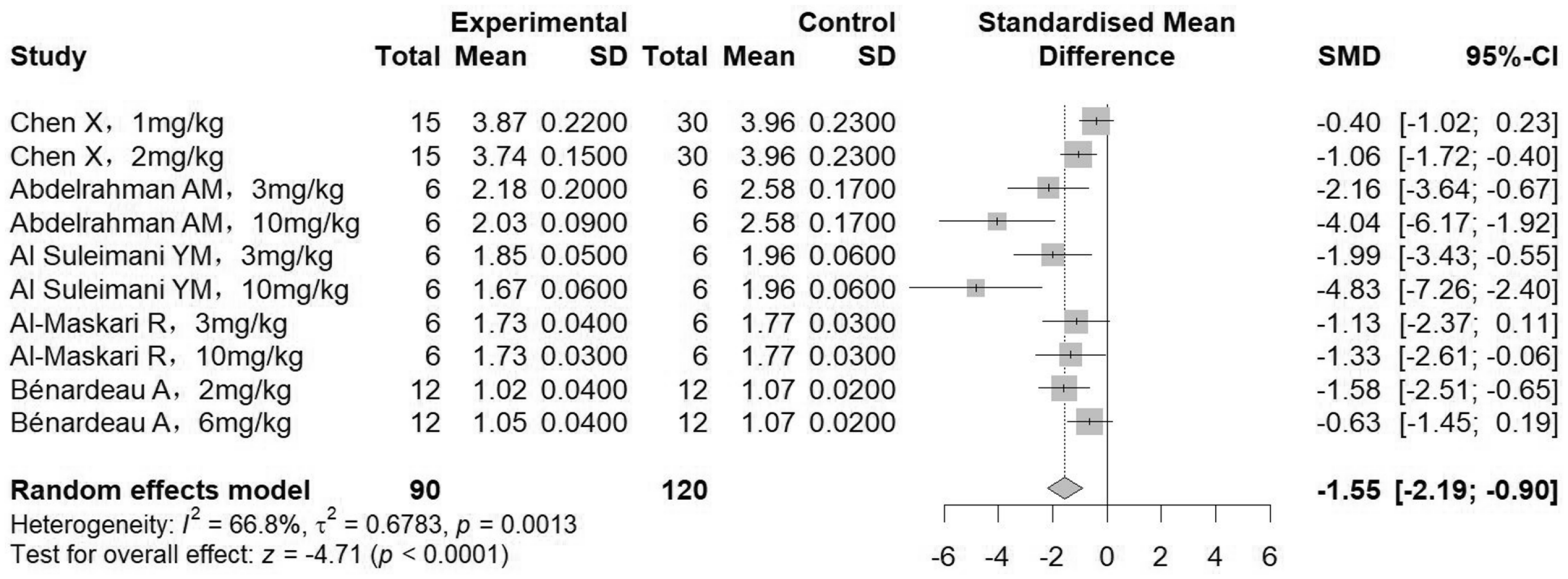
The effect of sGCs/sGCa treatment on kidney weight.

Body weight and kidney weight after treatment were reported in eight and five studies, respectively. Pooled analysis of eight studies (285 animals) revealed no significant difference in body weight between groups receiving sGCs/sGCa and control groups (SMD = −0.24, 95%CI: −1.17, 0.68, *p* = 0.604; *I^2^* = 84.6%) (Figure 2). In contrast, meta-analysis of five studies (210 animals) demonstrated a significant reduction in kidney weight in rodent models following treatment (SMD = −1.55, 95%CI: −2.19, −0.90, *p* < 0.001; *I^2^* = 66.8%) (Figure 3). Subgroup analysis further showed that this reduction in kidney weight occurred across various sGC classes and chronic kidney disease types (Table 3).

**Table 3:** Subgroup analyses for the comparison between outcomes.

Outcomes	NO	ES (95% CI)	P-within	*I*^2^ (%)	P-heterogeneity
Body weight					
Classification of sGC					
sGCs	11	−0.22 (−1.48, 1.05)	0.264	87.1	<0.001
sGCa	3	−0.47 (−1.34, 0.39)	0.102	66.4	0.051
Types of CKD					
Hypertensive nephropathy	7	−095 (−2.98, 1.08)	0.357	90.6	<0.001
Diabetic nephropathy	1	−1.35 (−2.31, −0.38)	0.006	NA	NA
Others	6	0.58 (0.10, 1.06)	0.017	0	0.869
Kidney weight					
Classification of sGC					
sGCs	8	−1.79 (−2.67, −0.91)	<0.001	71.7	0.001
sGCa	2	−1.08 (−2.01, −0.15)	0.001	55.6	0.134
Types of CKD					
Hypertensive nephropathy	4	−0.86 (−1.34, −0.38)	<0.001	40.6	0.168
Diabetic nephropathy	0	NA	NA	NA	NA
Others	6	−2.29 (−3.30, −1.27)	<0.001	57.5	0.038
Systolic blood pressure					
Classification of sGC					
sGCs	4	−7.30 (−13.59, −1.02)	<0.001	87.2	<0.001
sGCa	4	−0.91 (−1.65, −0.17)	0.001	60.3	0.056
Types of CKD					
Hypertensive nephropathy	3	−1.92 (−3.24, −0.60)	<0.001	87.1	0.001
Diabetic nephropathy	1	−0.10 (−0.97, 0.76)	0.816	NA	NA
Others	4	−6.88 (−13.79, 0.03)	0.051	89.5	<0.001
Serum creatinine					
Classification of sGC					
sGCs	11	−2.96 (−4.91, −1.00)	0.003	92.5	<0.001
sGCa	4	−4.05 (−7.82, −0.28)	0.035	92.7	<0.001
Types of CKD					
Hypertensive nephropathy	3	−1.21 (−5.56, 3.15)	0.587	95.2	<0.001
Diabetic nephropathy	4	−0.93 (−1.43, −0.44)	<0.001	0	0.678
Others	8	−5.37 (−7.46, −3.29)	<0.001	87.0	<0.001
Blood urea nitrogen					
Classification of sGC					
sGCs	9	−4.00 (−6.03, −1.96)	<0.001	87.7	<0.001
sGCa	4	−2.50 (−6.14, 1.14)	0.178	94.8	<0.001
Types of CKD					
Hypertensive nephropathy	3	−2.44 (−5.61, 0.73)	0.101	87.3	<0.001
Diabetic nephropathy	2	0.51 (−0.13, 1.14)	0.116	0	0.959
Others	8	−5.03 (−6.98, −3.08)	<0.001	51	0.057
Serum uric acid					
Classification of sGC					
sGCs	8	−3.82 (−4.84, −2.80)	<0.001	51.6	0.044
sGCa	0	NA	NA	NA	NA
Types of CKD					
Hypertensive nephropathy	0	NA	NA	NA	NA
Diabetic nephropathy	2	−4.50 (−5.88, −3.12)	<0.001	0	0.55
Others	6	−3.64 (−4.95, −2.33)	<0.001	56.9	0.041

#### 3.3.2 Assessment of systolic blood pressure

**Figure 4:**
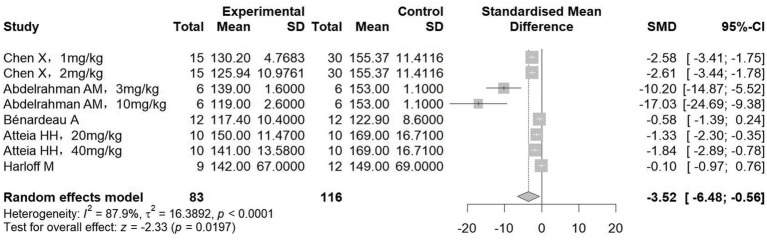
The effect of sGCs/sGCa treatment on systolic blood pressure.

Hypertension is intricately linked to chronic renal failure, serving as both a common etiology and a frequent complication. Analysis of blood pressure outcomes from five studies (involving 199 animals) elucidated a significant lowering effect in the treatment group relative to controls (SMD = −3.52, 95%CI: −6.48, −0.56, *p* = 0.019; *I^2^* = 87.9%) (Figure 4). Subgroup analysis showed that sGC classes and hypertension nephropathy models were both effective in reducing blood pressure; notably, there was no statistically significant change in blood pressure in diabetic nephropathy and other types of nephropathy models (Table 3).

#### 3.3.3 Assessment of serum creatinine and blood urea nitrogen

**Figure 5:**
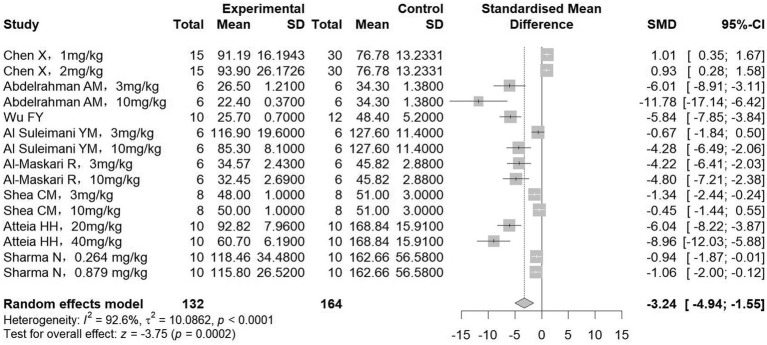
The effect of sGCs/sGCa treatment on serum creatinine.

**Figure 6:**
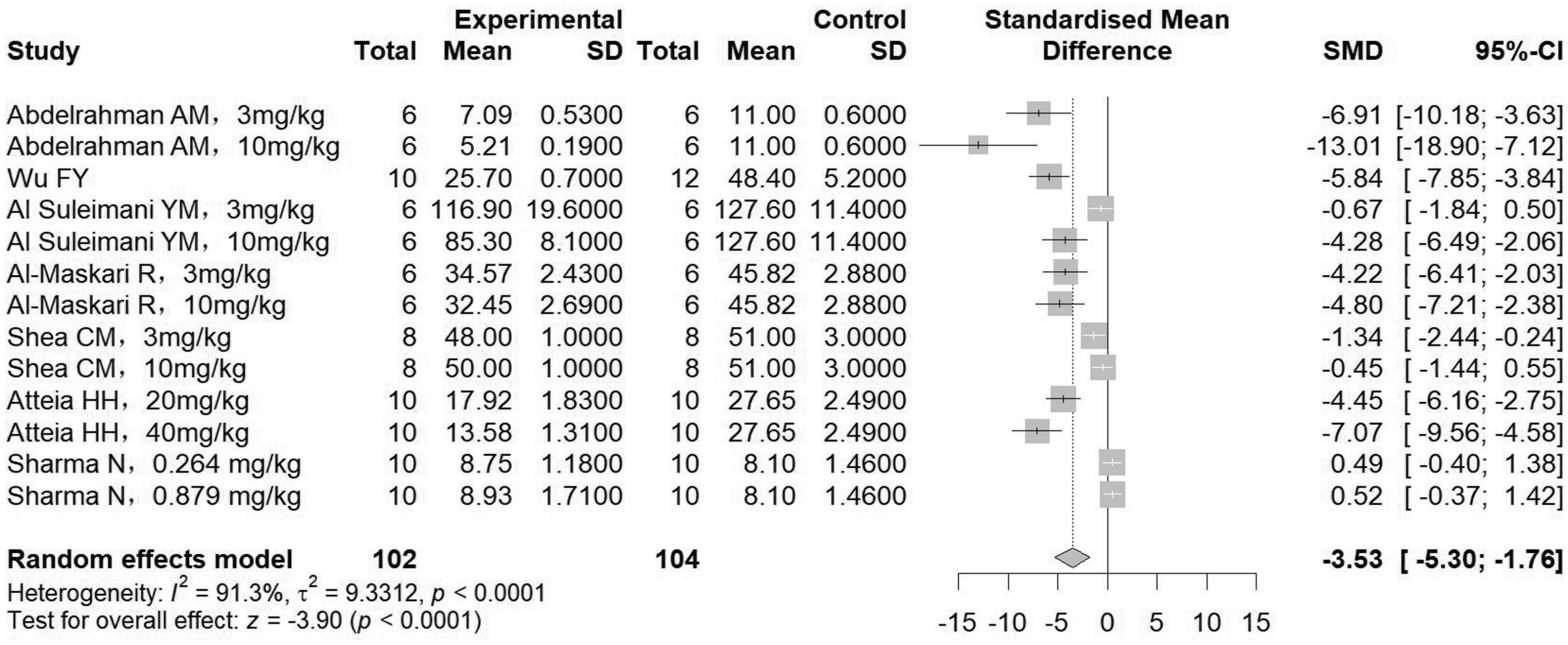
The effect of sGCs/sGCa treatment on blood urea nitrogen.

A total of 8 studies measured SCr (132 treatment, 164 control animals), and 7 studies measured BUN (102 treatment, 104 control animals). Meta-analysis showed that treatment significantly reduced both SCr and BUN levels compared to controls (SCr: SMD = −3.24, 95%CI: −4.94, −1.55, *p* < 0.001; *I^2^* = 92.6%; BUN: SMD = −3.53, 95%CI: −5.30, −1.76, *p* < 0.001; *I^2^* = 91.3%) (Figures 5, 6). Subgroup analyses, however, indicated no significant association between the reduction in BUN and the diabetic nephropathy and hypertensive nephropathy models (Table 3).

#### 3.3.4 Assessment of serum uric acid

**Figure 7:**
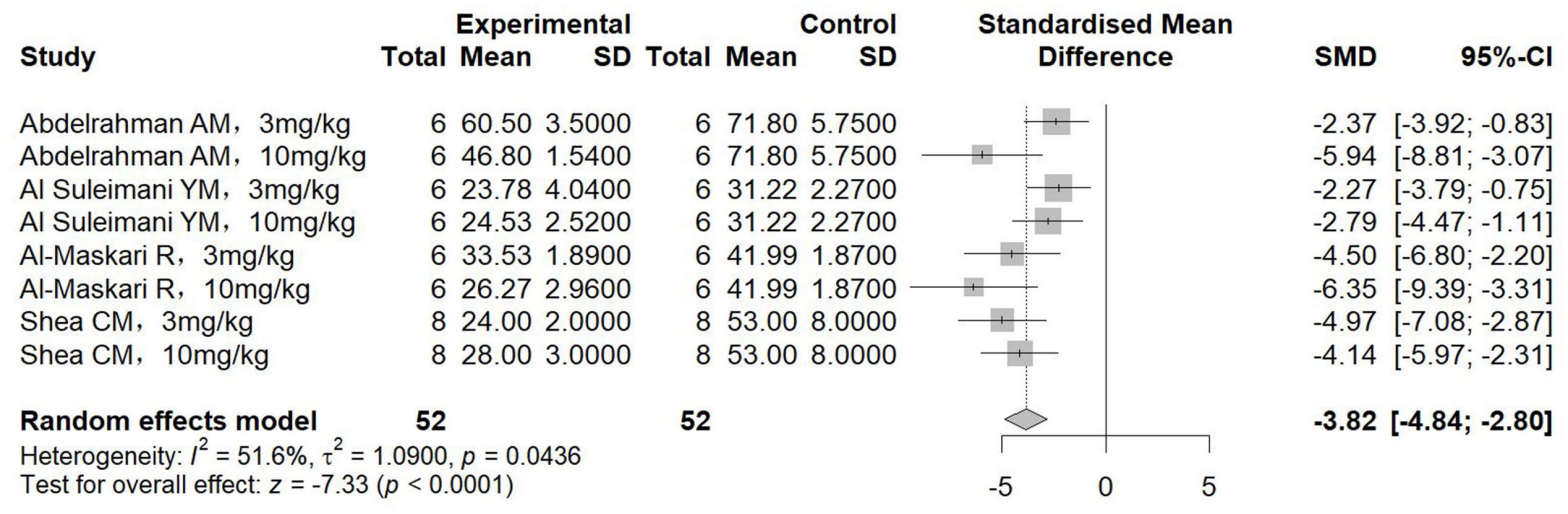
The effect of sGCs/sGCa treatment on serum uric acid.

Eight studies measuring serum uric acid (all involving sGCs) were included, with 52 animals assigned to both the treatment and control groups. The results showed a significant reduction in serum uric acid levels following treatment (SMD = −3.82, 95%CI: −4.84, −2.80, *p* < 0.001; *I^2^* = 51.6%) (Figure 7); Subgroup analysis further revealed no significant influence of the underlying sGC classes and chronic kidney disease types on this effect (Table 3).

### 3.4 Sensitivity analysis and publication bias

The overall pooled results were robust to sensitivity analyses (leave-one-out method) across all endpoints (body weight, kidney weight, SBP, SCr, BUN, and SUA) (Supplementary Figures 1–6).

Funnel plots along with Egger’s tests were used to evaluate publication bias. The results exhibited evidence of bias for kidney weight, SCr, BUN, and SUA (all *p* < 0.001), although trim-and-fill analysis indicated no substantial imputation. Conversely, the funnel plots for body weight (*p* = 0.747) and SBP (*p* = 0.057) demonstrated no marked asymmetry (Supplementary Figures 7–12).

## 4 Discussion

This systematic review and meta-analysis comprehensively evaluates the overall efficacy of sGCs/sGCa in various CKD animal models. The treatment exerts multifaceted benefits, including reduced kidney weight, lowered systolic blood pressure, improved renal function, and decreased serum uric acid levels. These results underscore the preclinical therapeutic potential of sGCs/sGCa in mitigating CKD progression and its complications.

Collectively, the results presented in Figures 2–7 highlight the multifaceted renoprotective effects of sGC stimulators and activators in CKD models. The absence of significant changes in body weight suggests a limited impact on general metabolic status, whereas reductions in kidney weight may reflect attenuation of renal hypertrophy or fibrotic remodeling. The blood pressure–lowering effects observed primarily in hypertensive nephropathy models underscore the role of sGC signaling in vascular regulation. Improvements in serum creatinine and blood urea nitrogen indicate preservation of renal excretory function, while the reduction in serum uric acid suggests potential benefits in correcting CKD-associated metabolic disturbances. Together, these findings support the biological plausibility of sGC modulation as a therapeutic strategy in CKD, while also highlighting disease-specific variability in treatment response.

sGC is the core enzyme mediating the NO-cGMP signaling pathway, which is crucial for maintaining vascular homeostasis (8). In the kidneys, activation of this pathway can induce afferent arteriolar dilation, reduce vascular resistance, and alleviate tubulointerstitial fibrosis (15, 16). However, in the state of chronic kidney disease (CKD), oxidative stress and chronic inflammation lead to uncoupling of the NO-cGMP signaling pathway, resulting in reduced NO synthesis and insufficient cGMP production, thereby accelerating disease progression (17, 18). The pharmacological action of sGCs/sGCa specifically targets this dysfunctional pathway to re-establish its activity. While existing clinical studies have mainly explored their use in diabetic nephropathy, our results reveal that these agents are equally effective in improving renal function and decelerating disease progression in hypertensive nephropathy and other CKD variants, thereby broadening the potential therapeutic landscape.

Substantial heterogeneity was observed for all endpoints. Consequently, subgroup analyses were conducted to examine the influence of sGC class and CKD classification on treatment effects. CKD etiology emerged as a significant contributor to heterogeneity, notably affecting systolic blood pressure (SBP) and blood urea nitrogen (BUN). The reduction in SBP was most marked in models of hypertensive nephropathy; however, the effect on BUN was notably attenuated in diabetic nephropathy. These findings suggest that the therapeutic response to sGCs/sGCa may be modulated by disease-specific pathophysiological mechanisms.

Nevertheless, this study has several limitations. First, variability in animal models and dosing regimens among the included studies introduces intrinsic heterogeneity. Second, the current evidence is predominantly based on short-term interventions, leaving long-term efficacy and safety largely unexplored. Furthermore, while subgroup analyses suggest that treatment effects may differ by CKD type, these findings require validation through more targeted investigations. Moreover, outcome reporting across studies was limited: only two included studies reported biomarker data, specifically neutrophil gelatinase-associated lipocalin (NGAL), precluding biomarker-based quantitative synthesis, and clinically relevant prognostic indicators such as GFR, CKD staging, and survival outcomes were insufficiently reported, restricting a more comprehensive evaluation of treatment efficacy and prognosis. Therefore, future large-scale clinical studies are required to determine the efficacy, safety, and optimal use of sGCs/sGCa in patients with CKD, in order to guide clinical practice with stronger evidence.

From a translational perspective, these findings highlight the clinical potential of sGC stimulators and activators as emerging therapeutic options for CKD. The observed variability across CKD subtypes further emphasizes the importance of patient stratification in future clinical trials. Prospective studies should therefore focus on well-defined CKD populations and incorporate clinically relevant endpoints to clarify the efficacy, safety, and long-term benefits of sGC-targeted therapies.

## 5 Conclusion

Our findings indicate that sGC stimulators and activators confer therapeutic benefits in rodent models of CKD, as reflected by reductions in kidney weight and systolic blood pressure, along with improvements in renal function and serum uric acid levels. However, the limited number of studies, heterogeneous outcome measures, and the absence of biomarker-based outcomes and data from target species restrict the robustness and translational relevance of the evidence. Nevertheless, the available data support the preclinical therapeutic potential of sGC modulators and justify further investigation.

## Data Availability

The original contributions presented in the study are included in the article/Supplementary material, further inquiries can be directed to the corresponding author.
