# Supplementary material for: Efficacy and safety of soluble guanylate cyclase stimulators or activators for chronic kidney disease: a systematic review and meta-analysis
Source: Front Med (Lausanne). 2026 Mar 2;13:1778037. doi: 10.3389/fmed.2026.1778037 (PMC12989487; doi:10.3389/fmed.2026.1778037)
Supplement: Supplementary file 1 [file Supplementary_file_1.docx]

**Supplementary Table 1. Search strategy in databases**

**Pubmed**

1. (("solubility"[MeSH Terms] OR "solubility"[All Fields] OR "solubilities"[All Fields] OR "soluble"[All Fields] OR "solubles"[All Fields] OR "solublization"[All Fields] OR "solublize"[All Fields] OR "solublized"[All Fields]) AND ("guanyl"[All Fields] OR "guanylate"[All Fields] OR "guanylates"[All Fields] OR "guanylic"[All Fields]) AND ("stimulate"[All Fields] OR "stimulated"[All Fields] OR "stimulates"[All Fields] OR "stimulating"[All Fields] OR "stimulation"[All Fields] OR "stimulations"[All Fields] OR "stimulative"[All Fields] OR "stimulator"[All Fields] OR "stimulator s"[All Fields] OR "stimulators"[All Fields])) OR (("solubility"[MeSH Terms] OR "solubility"[All Fields] OR "solubilities"[All Fields] OR "soluble"[All Fields] OR "solubles"[All Fields] OR "solublization"[All Fields] OR "solublize"[All Fields] OR "solublized"[All Fields]) AND ("guanyl"[All Fields] OR "guanylate"[All Fields] OR "guanylates"[All Fields] OR "guanylic"[All Fields]) AND ("activable"[All Fields] OR "activate"[All Fields] OR "activated"[All Fields] OR "activates"[All Fields] OR "activating"[All Fields] OR "activation"[All Fields] OR "activations"[All Fields] OR "activator"[All Fields] OR "activator s"[All Fields] OR "activators"[All Fields] OR "active"[All Fields] OR "actived"[All Fields] OR "actively"[All Fields] OR "actives"[All Fields] OR "activities"[All Fields] OR "activity s"[All Fields] OR "activitys"[All Fields] OR "exercise"[MeSH Terms] OR "exercise"[All Fields] OR "activity"[All Fields])) OR ("riociguat"[Supplementary Concept] OR "riociguat"[All Fields]) OR ("vericiguat"[Supplementary Concept] OR "vericiguat"[All Fields]) OR ("praliciguat"[Supplementary Concept] OR "praliciguat"[All Fields]) OR ("bay 58 2667"[Supplementary Concept] OR "bay 58 2667"[All Fields] OR "cinaciguat"[All Fields])
2. "Chronic Kidney Disease"[All Fields] OR "Chronic Kidney Diseases"[All Fields] OR "Chronic Kidney Insufficiency"[All Fields] OR "Chronic Renal Disease"[All Fields] OR "Chronic Renal Diseases"[All Fields] OR "Chronic Renal Insufficiencies"[All Fields] OR "Chronic Renal Insufficiency"[All Fields] OR "disease chronic kidney"[All Fields] OR "disease chronic renal"[All Fields] OR "diseases chronic kidney"[All Fields] OR "diseases chronic renal"[All Fields] OR "kidney disease chronic"[All Fields] OR "kidney diseases chronic"[All Fields] OR "kidney insufficiency chronic"[All Fields] OR "renal disease chronic"[All Fields] OR "renal diseases chronic"[All Fields] OR "renal insufficiencies chronic"[All Fields]
3. #1 AND #2

**Embase**

1. chronic kidney diseases':ti,ab,kw OR 'chronic kidney insufficiency':ti,ab,kw OR 'chronic renal disease':ti,ab,kw OR 'chronic renal diseases':ti,ab,kw OR 'chronic renal insufficiencies':ti,ab,kw OR 'chronic renal insufficiency':ti,ab,kw OR 'disease, chronic kidney':ti,ab,kw OR 'disease, chronic renal':ti,ab,kw OR 'diseases, chronic kidney':ti,ab,kw OR 'diseases, chronic renal':ti,ab,kw OR 'kidney disease, chronic':ti,ab,kw OR 'kidney diseases, chronic':ti,ab,kw OR 'kidney insufficiency, chronic':ti,ab,kw OR 'renal disease, chronic':ti,ab,kw OR 'renal diseases, chronic':ti,ab,kw OR 'renal insufficiencies, chronic':ti,ab,kw.
2. soluble guanylate stimulators':ti,ab,kw OR soluble guanylate activators':ti,ab,kw OR riociguat:ti,ab,kw OR vericiguat:ti,ab,kw OR praliciguat:ti,ab,kw OR cinaciguat:ti,ab,kw.
3. #1 AND #2

**Web of Science**

1. Chronic Kidney Disease (Topic) OR Chronic Kidney Diseases (Topic) OR Chronic Kidney Insufficiency (Topic) OR Chronic Renal Disease (Topic) OR Chronic Renal Diseases (Topic) OR Chronic Renal Insufficiencies (Topic) OR Chronic Renal Insufficiency (Topic) OR Disease, Chronic Kidney (Topic) OR Disease, Chronic Renal (Topic) OR Diseases, Chronic Kidney (Topic) OR Diseases, Chronic Renal (Topic) OR Kidney Disease, Chronic (Topic) OR Kidney Diseases, Chronic (Topic) OR Kidney Insufficiency, Chronic (Topic) OR Renal Disease, Chronic (Topic) OR Renal Diseases, Chronic (Topic) OR Renal Insufficiencies, Chronic (Topic)
2. soluble guanylate stimulators (Topic) OR soluble guanylate activators (Topic) OR riociguat (Topic) OR vericiguat (Topic) OR praliciguat (Topic) OR cinaciguat (Topic)
3. #1 AND #2

**Cochrane Library**

1. ((("Chronic Kidney Disease" OR "Chronic Kidney Diseases" OR "Chronic Kidney Insufficiency" OR "Chronic Renal Disease" OR "Chronic Renal Diseases" OR "Chronic Renal Insufficiencies" OR "Chronic Renal Insufficiency" OR "Disease, Chronic Kidney" OR "Disease, Chronic Renal" OR "Diseases, Chronic Kidney" OR "Diseases, Chronic Renal" OR "Kidney Disease, Chronic" OR "Kidney Diseases, Chronic" OR "Kidney Insufficiency, Chronic" OR "Renal Disease, Chronic" OR "Renal Diseases, Chronic" OR "Renal Insufficiencies, Chronic"))):ti,ab,kw
2. ((((((soluble guanylate stimulators) OR (soluble guanylate activators)) OR (riociguat)) OR (vericiguat)) OR (praliciguat)) OR (cinaciguat)):ti,ab,kw
3. #1 AND #2


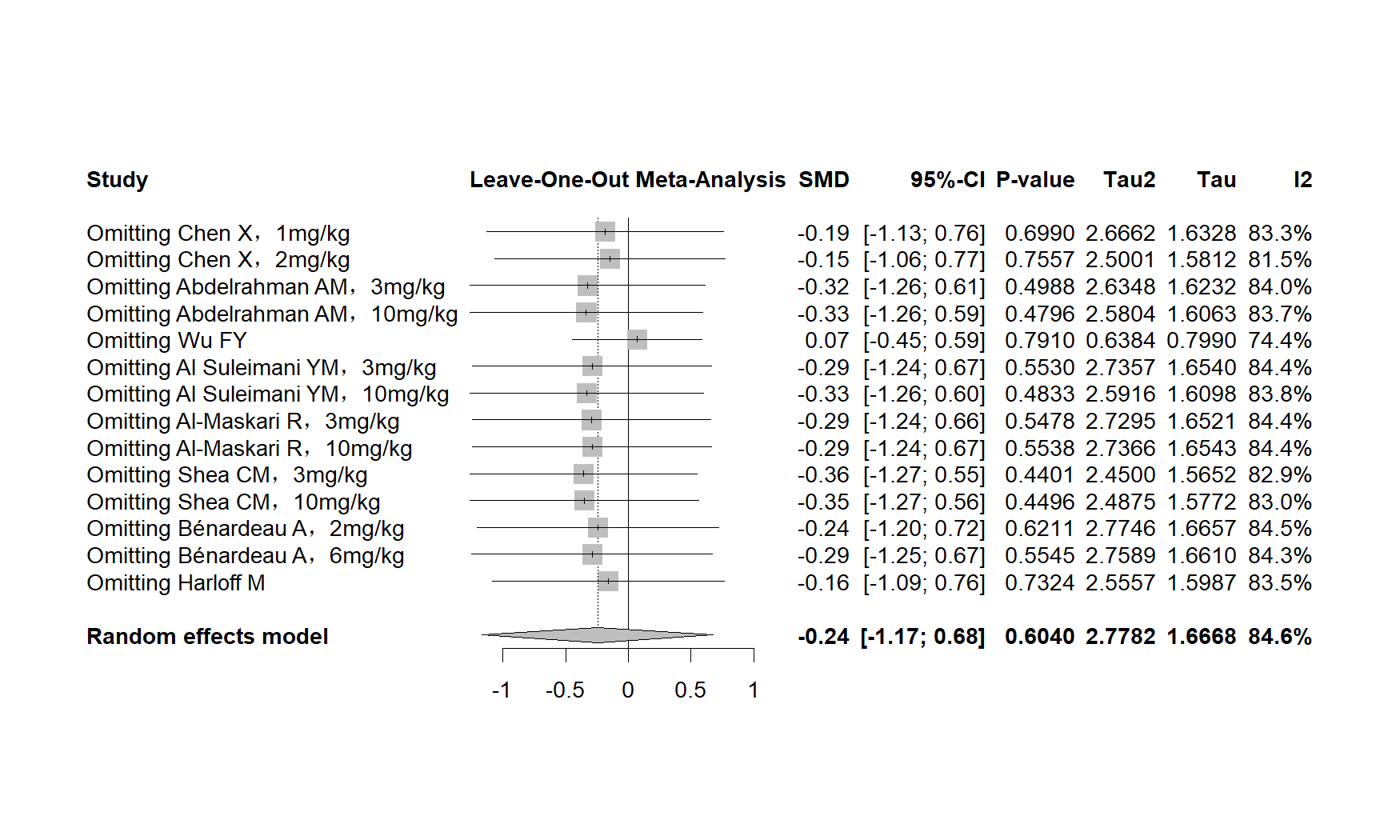


Supp Fig. 1. Sensitivity Analysis of sGCs/sGCa on body weight


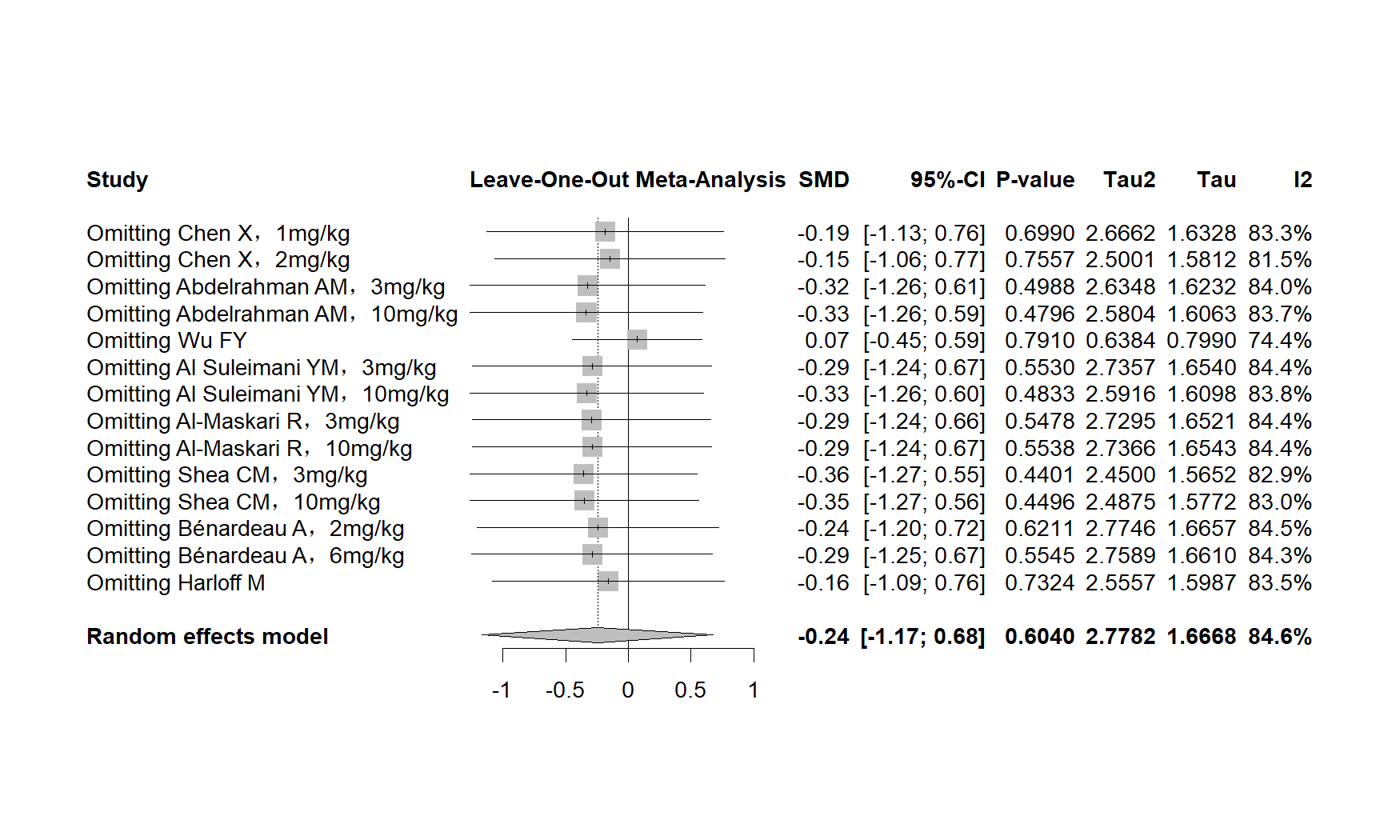


Supp Fig. 2. Sensitivity Analysis of sGCs/sGCa on kidney weight


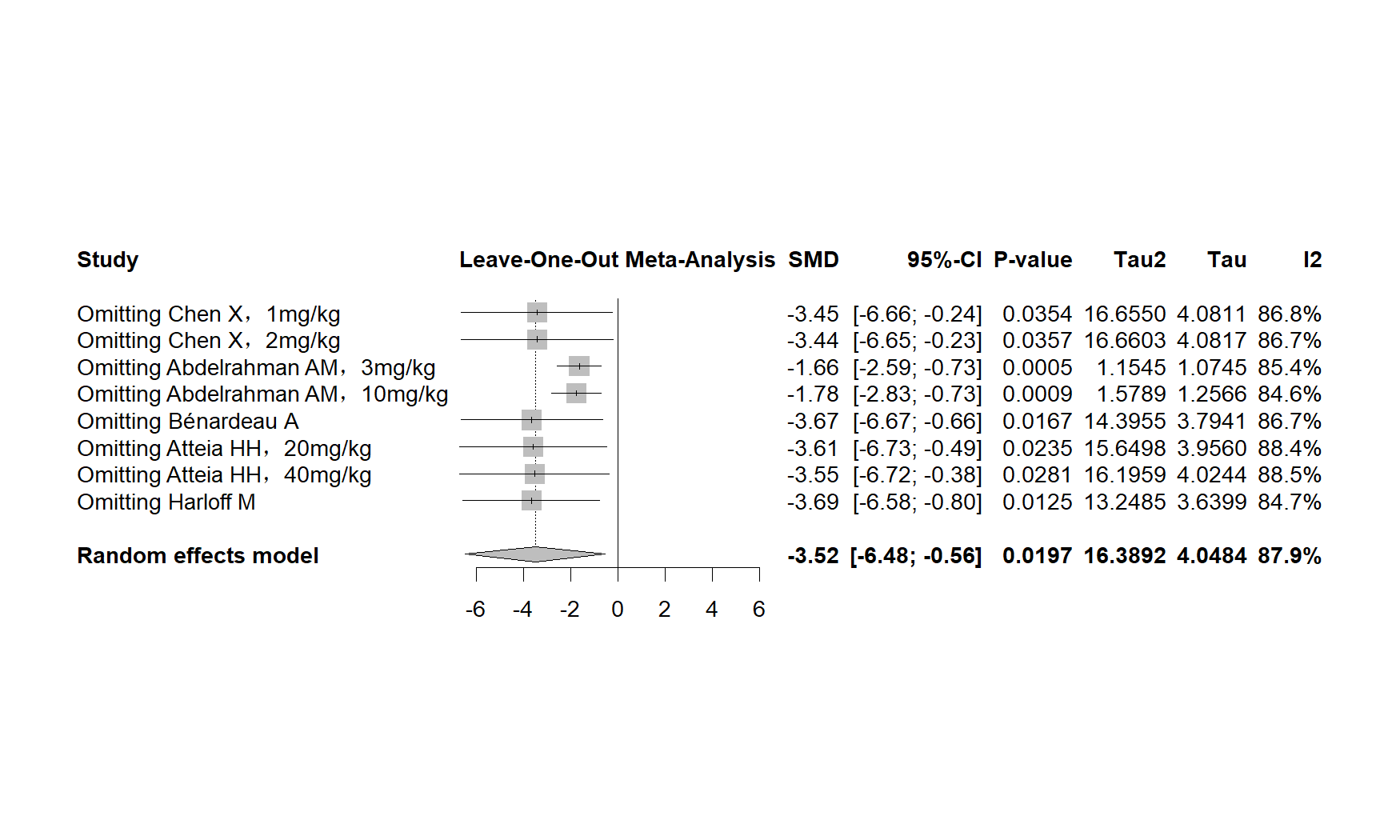


Supp Fig. 3. Sensitivity Analysis of sGCs/sGCa on SBP


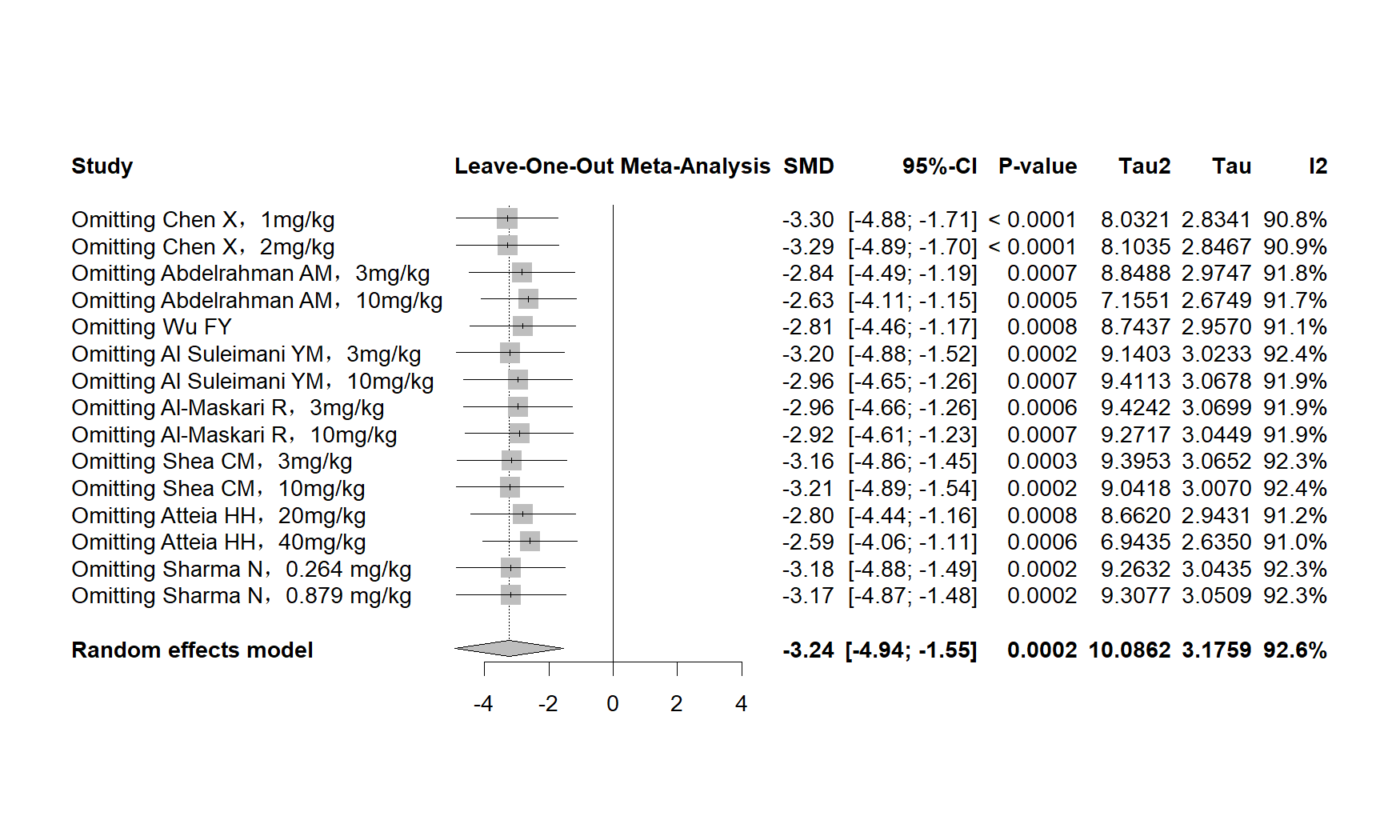


Supp Fig. 4. Sensitivity Analysis of sGCs/sGCa on SCr


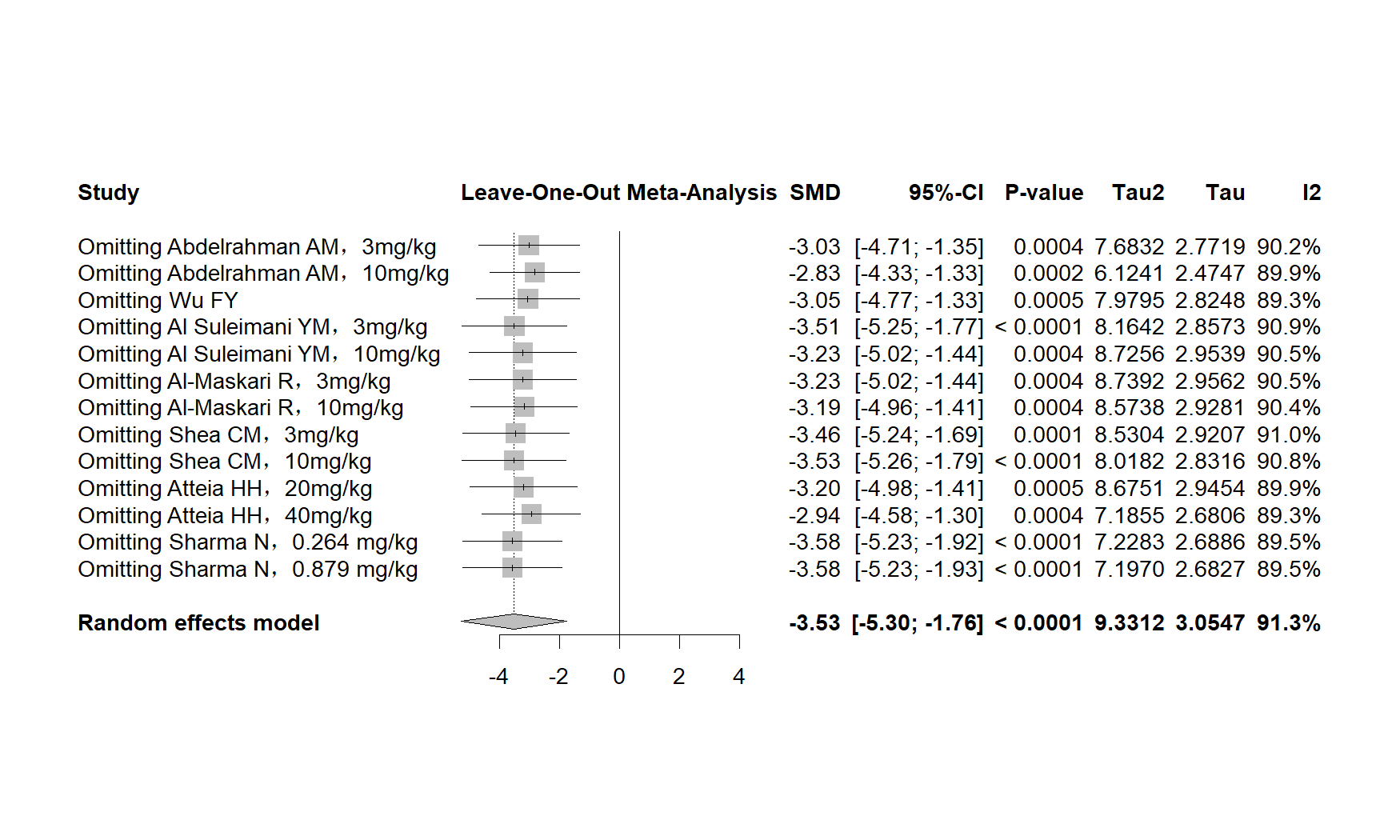


Supp Fig. 5. Sensitivity Analysis of sGCs/sGCa on BUN


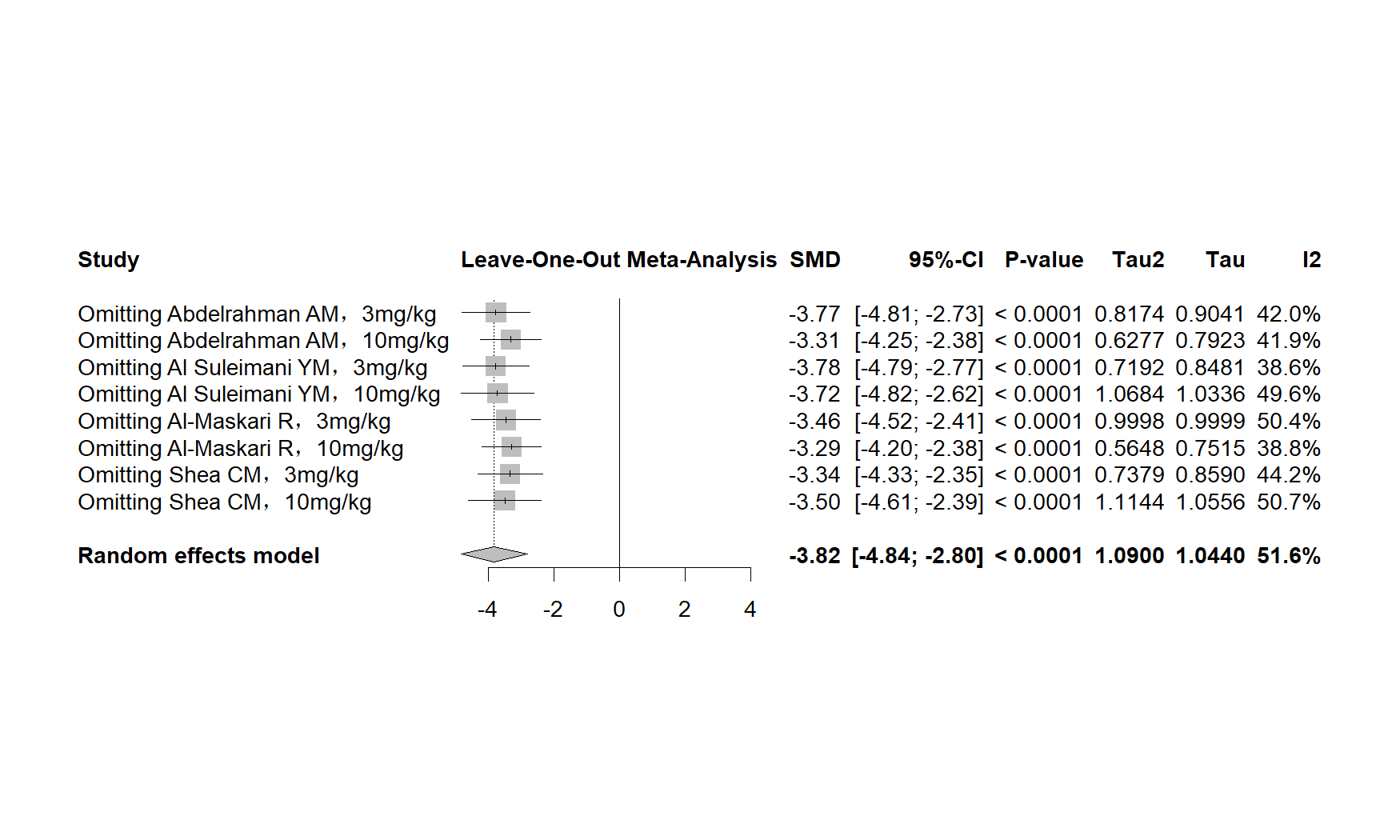


Supp Fig. 6. Sensitivity Analysis of sGCs/sGCa on SUA


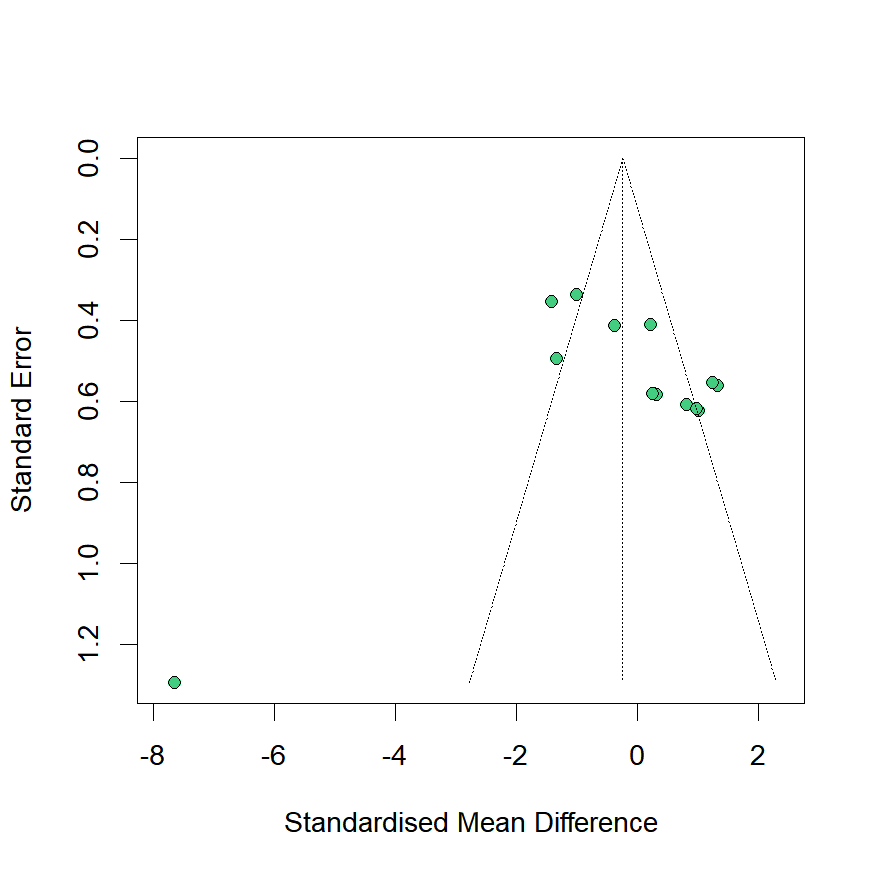

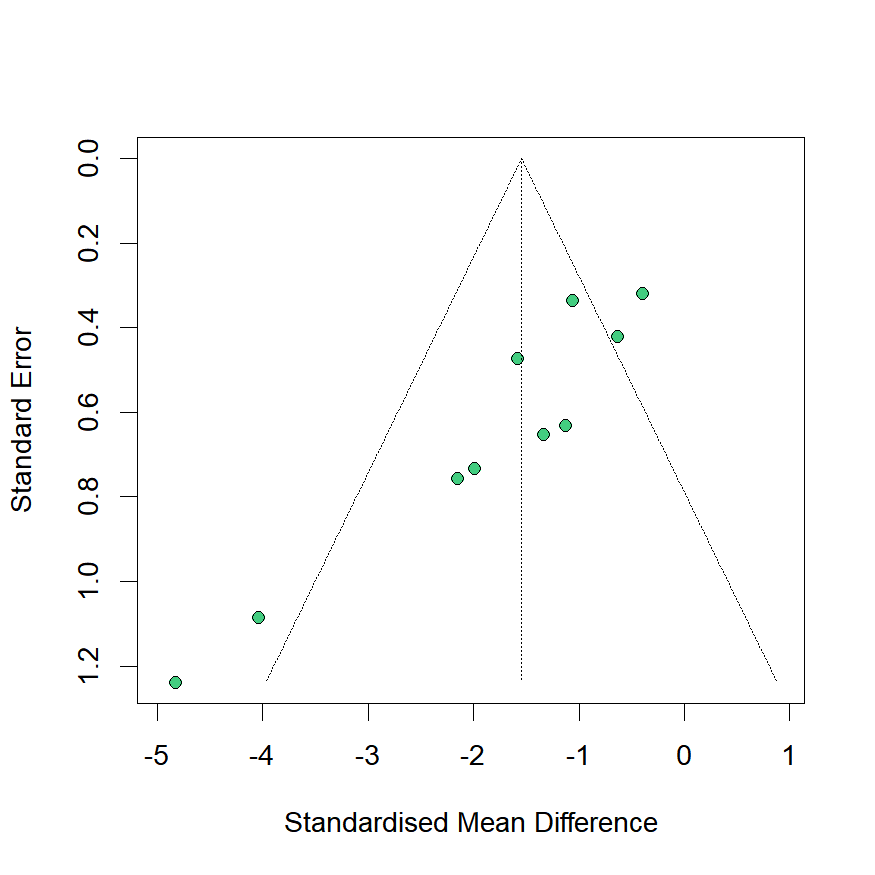


Supp Fig. 7. Funnel plots for body weight Supp Fig. 8. Funnel plots for kidney weight


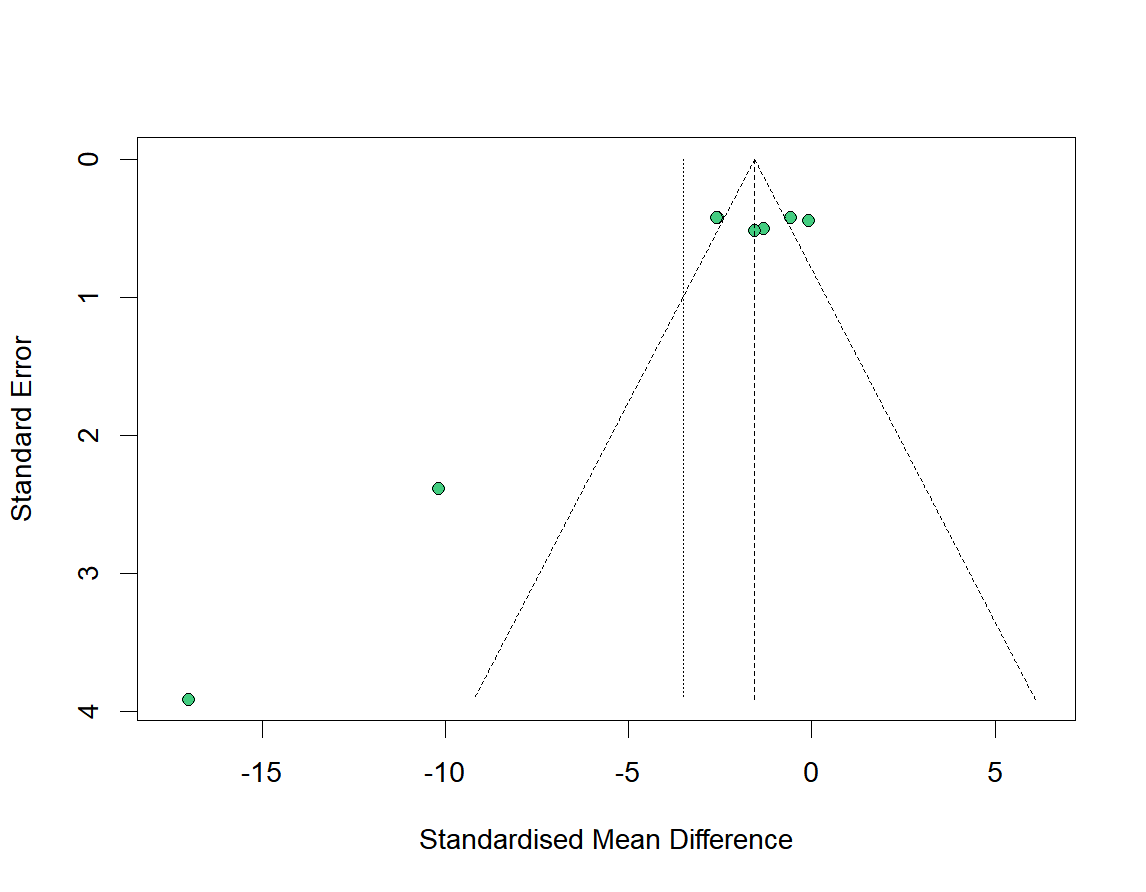


Supp Fig. 9. Funnel plots for SBP


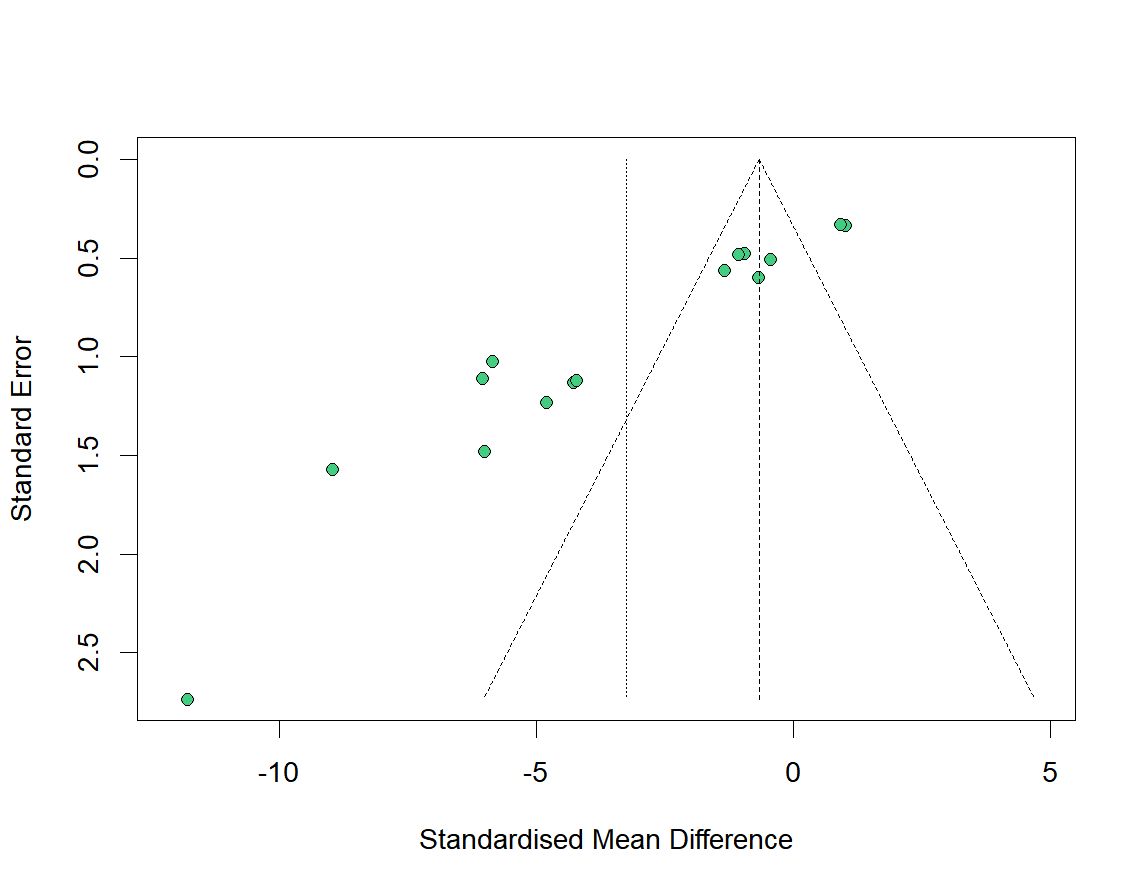


Supp Fig. 10. Funnel plots for SCr


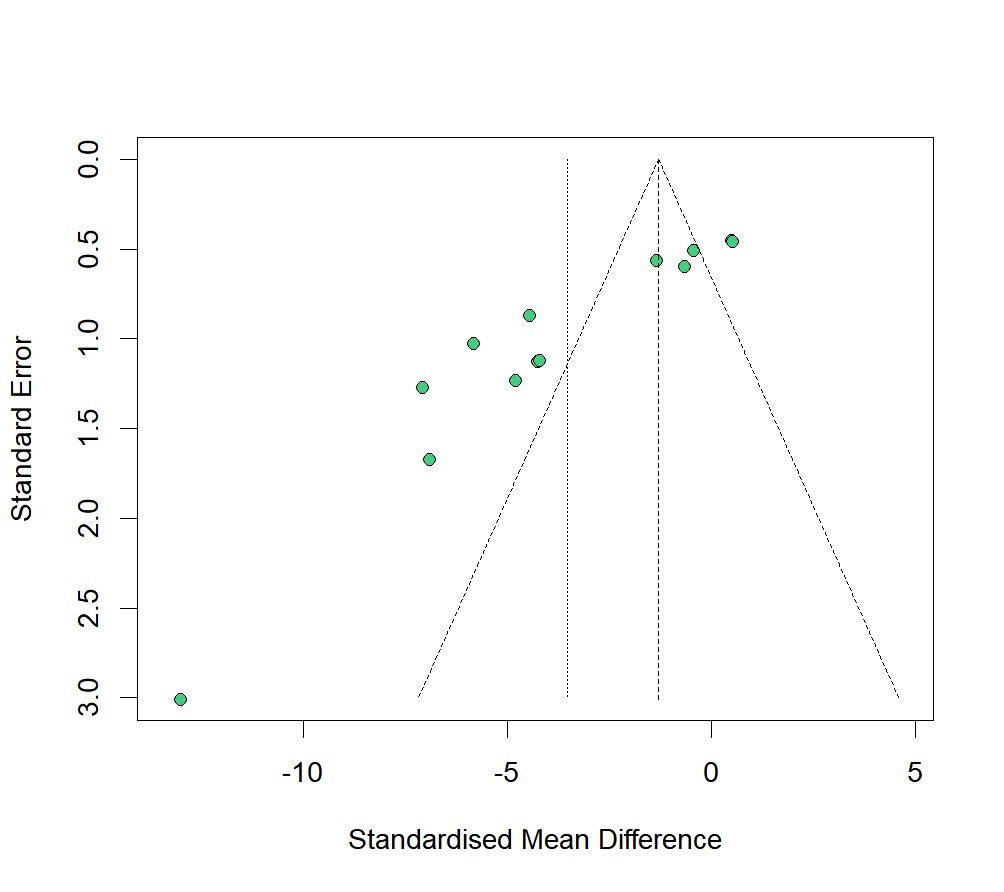


Supp Fig. 11. Funnel plots for BUN


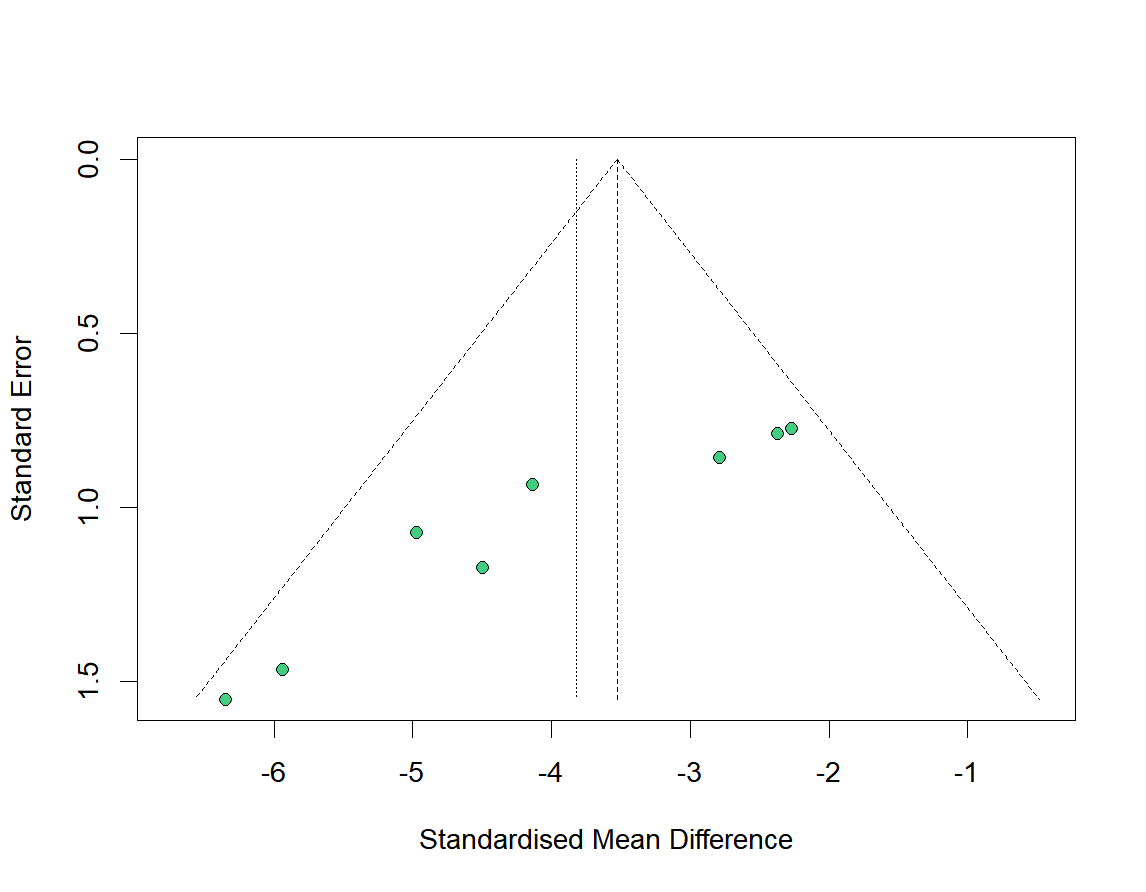


Supp Fig. 12. Funnel plots for SUA
